# Supplementary material for: Dysregulated naive B cells and de novo autoreactivity in severe COVID-19
Source: Nature. 2022 Aug 31;611(7934):139–47. doi: 10.1038/s41586-022-05273-0 (PMC9630115; doi:10.1038/s41586-022-05273-0)
Supplement: Supplementary file 1 — A guide for Supplementary Tables 1–4 (tables supplied separately). [file 41586_2022_5273_MOESM1_ESM.pdf]

nature portfolio

https://doi.org/10.1038/s41586-022-05273-0

Supplementary information

Dysregulated naive B cells and de novo autoreactivity in severe COVID-19

In the format provided by the
authors and unedited

Dysregulated naïve B cells and de novo autoreactivity in severe COVID-19

Authors: Matthew C. Woodruff[1,2], Richard P. Ramonell[3], Natalie S. Haddad[4], Fabliha A. Anam[1,2], Mark E. Rudolph[5], Tiffany A. Walker[6], Alexander D. Truong[7], Adviteeya N. Dixit[7], Jenny E. Han[6], Monica Cabrera-Mora[7], Martin C. Runnstrom[7], Regina Bugrovsky[1,2], Jennifer Hom[1,2], Erin C. Connolly[8], Igor Albizua[9], Vidhi Javia[7], Kevin S. Cashman[1,2], Doan C. Nguyen[7], Shuya Kyu[7], Ankur Singh Saini[1,2], Michael Piazza[10], Christopher M. Tipton[1,2], Arezou Khosroshahi[1,2], Greg Gibson[8], Greg S. Martin[7], Cheryl L. Maier[9], Annette Esper[7], Scott A. Jenks[1,2], F. Eun-Hyung Lee*[7], Ignacio Sanz*[1,2].

[1]Department of Medicine, Division of Rheumatology, Lowance Center for Human Immunology, Emory University, Atlanta, GA, USA

[2]Emory Autoimmunity Center of Excellence, Emory University, Atlanta, GA, USA

[3]Department of Medicine, Division of Pulmonary, Allergy and Critical Care Medicine, University of Pittsburgh, Pittsburgh, PA, USA

[4]MicroB-plex, Atlanta, GA, USA

[5]Exagen Inc., Vista, CA, USA

[6]Department of Medicine, Division of General Internal Medicine, Emory University, Atlanta, GA, USA

[7]Department of Medicine, Division of Pulmonary, Allergy, Critical Care and Sleep Medicine, Emory University, Atlanta, GA, USA

[8]School of Biological Sciences, Georgia Institute of Technology, Atlanta, GA, USA.

[9]Department of Pathology and Laboratory Medicine, Center for Transfusion and Cellular Therapies, Emory University School of Medicine, Emory University, Atlanta, GA, USA

[10]Nicoya, Kitchener-Waterloo, Canada

These authors contributed equally to this work: Matthew C. Woodruff and Richard P. Ramonell

*Corresponding Authors
F. Eun-Hyung Lee – F.E.Lee@emory.edu
Ignacio Sanz – Ignacio.sanz@emory.edu

Table of Contents:

Supplemental table 1 – Patient/donor demographics

Supplemental table 2 – Antibody catalogue and panel design

Supplemental table 3 – Detailed patient demographics for single cell analysis

Supplemental table 4 – Monoclonal antibody details and screening results

Supplemental table 1 – Patient/donor demographics (Supplemental tables, tab 1)
An overview of patient demographics included in the study by disease cohort

Supplemental table 2 – Antibody catalogue and panel design (Supplemental tables, tab 2)
A complete catalogue of antibodies used to classify and sort B cell populations as in Extended data figure 1

Supplemental table 3 – Detailed patient demographics for single cell analysis (Supplemental tables, tab 3)
Individual demographics of patients included for single cell repertoire analysis. Included are single cell library counts, clonotypes, and clonality statistics.

Supplemental table 4 – Monoclonal antibody details and screening results (Supplemental tables, tab 4)
Detailed characterizations of monoclonal antibodies identified and tested. Includes heavy and light chain identities, B cell population inclusion, and reactivity against tested viral and self-antigens.
