# Supplementary material for: Dysregulated naive B cells and de novo autoreactivity in severe COVID-19
Source: Nature. 2022 Aug 31;611(7934):139–47. doi: 10.1038/s41586-022-05273-0 (PMC9630115; doi:10.1038/s41586-022-05273-0)
Supplement: Supplementary file 2 — Reporting Summary [file 41586_2022_5273_MOESM2_ESM.pdf]

nature portfolio

Corresponding author(s): Dr. Ignacio Sanz

Last updated by author(s): 08/15/2022

Reporting Summary

Nature Portfolio wishes to improve the reproducibility of the work that we publish. This form provides structure for consistency and transparency in reporting. For further information on Nature Portfolio policies, see our Editorial Policies and the Editorial Policy Checklist.

Statistics

For all statistical analyses, confirm that the following items are present in the figure legend, table legend, main text, or Methods section.

n/a	Confirmed	
☐	☒	The exact sample size (*n*) for each experimental group/condition, given as a discrete number and unit of measurement
☐	☒	A statement on whether measurements were taken from distinct samples or whether the same sample was measured repeatedly
☐	☒	The statistical test(s) used AND whether they are one- or two-sided<br>*Only common tests should be described solely by name; describe more complex techniques in the Methods section.*
☒	☐	A description of all covariates tested
☒	☐	A description of any assumptions or corrections, such as tests of normality and adjustment for multiple comparisons
☐	☒	A full description of the statistical parameters including central tendency (e.g. means) or other basic estimates (e.g. regression coefficient) AND variation (e.g. standard deviation) or associated estimates of uncertainty (e.g. confidence intervals)
☐	☒	For null hypothesis testing, the test statistic (e.g. *F*, *t*, *r*) with confidence intervals, effect sizes, degrees of freedom and *P* value noted<br>*Give P values as exact values whenever suitable.*
☒	☐	For Bayesian analysis, information on the choice of priors and Markov chain Monte Carlo settings
☒	☐	For hierarchical and complex designs, identification of the appropriate level for tests and full reporting of outcomes
☐	☒	Estimates of effect sizes (e.g. Cohen's *d*, Pearson's *r*), indicating how they were calculated

Our web collection on statistics for biologists contains articles on many of the points above.

Software and code

Policy information about availability of computer code

Data collection	Patient data was collected using RedCAP data capture software. Flow cytometry data was collected on a Cytek Aurora flow cytometer using Cytek SpectroFlo software. Luminex data (including antigen specific data) was analyzed using a Luminex FLEXMAP 3D® instrument (Luminex; Austin, TX, USA) running xPonent 4.3 software. Repertoire data was sequenced by Novogene, and then processed through the 10x VDJ repertoire pipeline. Resulting sequences of high confidence were then mapped using IMGT's V-quest B cell receptor mapping software.
Data analysis	Computational analysis was carried out in R (v3.6.2; release 12 Dec 2019). Heat maps were generated using the pheatmap library (v1.0.12), with data pre-normalized (log-transformed z-scores calculated per feature) before plotting. Custom plotting, such as mutation frequency violin plots, was performed using the ggplot2 library for base analysis, and then post-processed in Adobe Illustrator. Alluvial plotting was performed using the ggalluvial package with post-processing in Adobe Illustrator. Clonotype connectivity analysis was carried out using the R-based 'vegan' package, and then visualized through 'pheatmap' before post-processing in Adobe Illustrator. Statistical analyses were performed directly in R, or in GraphPad Prism (v8.2.1).<br><br>Analyses on the single cell VDJ annotated sequences were performed using the Immcantation tool suite (http://www.immcantation.org) version 4.1.0 pipeline in Docker. This suite contains SHazaM for statistical analysis of somatic hypermutation (SHM) patterns as described in (Gupta et al., 2015), and BASELINe (Bayesian estimation of Antigen-driven SELectIoN) for analysis of selection pressure as described in (Yaari et al., 2012). Visualizations were generated in R using the SHazaM package (version 1.0.2) and then post-processed in Adobe Illustrator.

For manuscripts utilizing custom algorithms or software that are central to the research but not yet described in published literature, software must be made available to editors and reviewers. We strongly encourage code deposition in a community repository (e.g. GitHub). See the Nature Portfolio guidelines for submitting code & software for further information.

Data

Policy information about availability of data

All manuscripts must include a data availability statement. This statement should provide the following information, where applicable:

- Accession codes, unique identifiers, or web links for publicly available datasets
- A description of any restrictions on data availability
- For clinical datasets or third party data, please ensure that the statement adheres to our policy

All FCM and sequencing data presented here are publicly available in alignment with current requirements for public disclosure before peer review. All FCM data presented and analyzed in this manuscript (Fig. 1) are publicly available in the FlowRepository at http://flowrepository.org/id/FR-FCM-Z2XF/.

Field-specific reporting

Please select the one below that is the best fit for your research. If you are not sure, read the appropriate sections before making your selection.

☐ Life sciences ☐ Behavioural & social sciences ☐ Ecological, evolutionary & environmental sciences

For a reference copy of the document with all sections, see nature.com/documents/nr-reporting-summary-flat.pdf

Life sciences study design

All studies must disclose on these points even when the disclosure is negative.

Sample size	*Describe how sample size was determined, detailing any statistical methods used to predetermine sample size OR if no sample-size calculation was performed, describe how sample sizes were chosen and provide a rationale for why these sample sizes are sufficient.*
Data exclusions	*Describe any data exclusions. If no data were excluded from the analyses, state so OR if data were excluded, describe the exclusions and the rationale behind them, indicating whether exclusion criteria were pre-established.*
Replication	*Describe the measures taken to verify the reproducibility of the experimental findings. If all attempts at replication were successful, confirm this OR if there are any findings that were not replicated or cannot be reproduced, note this and describe why.*
Randomization	*Describe how samples/organisms/participants were allocated into experimental groups. If allocation was not random, describe how covariates were controlled OR if this is not relevant to your study, explain why.*
Blinding	*Describe whether the investigators were blinded to group allocation during data collection and/or analysis. If blinding was not possible, describe why OR explain why blinding was not relevant to your study.*

Behavioural & social sciences study design

All studies must disclose on these points even when the disclosure is negative.

Study description	*Briefly describe the study type including whether data are quantitative, qualitative, or mixed-methods (e.g. qualitative cross-sectional, quantitative experimental, mixed-methods case study).*
Research sample	*State the research sample (e.g. Harvard university undergraduates, villagers in rural India) and provide relevant demographic information (e.g. age, sex) and indicate whether the sample is representative. Provide a rationale for the study sample chosen. For studies involving existing datasets, please describe the dataset and source.*
Sampling strategy	*Describe the sampling procedure (e.g. random, snowball, stratified, convenience). Describe the statistical methods that were used to predetermine sample size OR if no sample-size calculation was performed, describe how sample sizes were chosen and provide a rationale for why these sample sizes are sufficient. For qualitative data, please indicate whether data saturation was considered, and what criteria were used to decide that no further sampling was needed.*
Data collection	*Provide details about the data collection procedure, including the instruments or devices used to record the data (e.g. pen and paper, computer, eye tracker, video or audio equipment) whether anyone was present besides the participant(s) and the researcher, and whether the researcher was blind to experimental condition and/or the study hypothesis during data collection.*
Timing	*Indicate the start and stop dates of data collection. If there is a gap between collection periods, state the dates for each sample cohort.*
Data exclusions	*If no data were excluded from the analyses, state so OR if data were excluded, provide the exact number of exclusions and the rationale behind them, indicating whether exclusion criteria were pre-established.*
Non-participation	*State how many participants dropped out/declined participation and the reason(s) given OR provide response rate OR state that no participants dropped out/declined participation.*

Randomization	*If participants were not allocated into experimental groups, state so OR describe how participants were allocated to groups, and if allocation was not random, describe how covariates were controlled.*

Ecological, evolutionary & environmental sciences study design

All studies must disclose on these points even when the disclosure is negative.

Study description	*Briefly describe the study. For quantitative data include treatment factors and interactions, design structure (e.g. factorial, nested, hierarchical), nature and number of experimental units and replicates.*
Research sample	*Describe the research sample (e.g. a group of tagged Passer domesticus, all Stenocereus thurberi within Organ Pipe Cactus National Monument), and provide a rationale for the sample choice. When relevant, describe the organism taxa, source, sex, age range and any manipulations. State what population the sample is meant to represent when applicable. For studies involving existing datasets, describe the data and its source.*
Sampling strategy	*Note the sampling procedure. Describe the statistical methods that were used to predetermine sample size OR if no sample-size calculation was performed, describe how sample sizes were chosen and provide a rationale for why these sample sizes are sufficient.*
Data collection	*Describe the data collection procedure, including who recorded the data and how.*
Timing and spatial scale	*Indicate the start and stop dates of data collection, noting the frequency and periodicity of sampling and providing a rationale for these choices. If there is a gap between collection periods, state the dates for each sample cohort. Specify the spatial scale from which the data are taken*
Data exclusions	*If no data were excluded from the analyses, state so OR if data were excluded, describe the exclusions and the rationale behind them, indicating whether exclusion criteria were pre-established.*
Reproducibility	*Describe the measures taken to verify the reproducibility of experimental findings. For each experiment, note whether any attempts to repeat the experiment failed OR state that all attempts to repeat the experiment were successful.*
Randomization	*Describe how samples/organisms/participants were allocated into groups. If allocation was not random, describe how covariates were controlled. If this is not relevant to your study, explain why.*
Blinding	*Describe the extent of blinding used during data acquisition and analysis. If blinding was not possible, describe why OR explain why blinding was not relevant to your study.*

Did the study involve field work? ☐ Yes ☐ No

Field work, collection and transport

Field conditions	*Describe the study conditions for field work, providing relevant parameters (e.g. temperature, rainfall).*
Location	*State the location of the sampling or experiment, providing relevant parameters (e.g. latitude and longitude, elevation, water depth).*
Access & import/export	*Describe the efforts you have made to access habitats and to collect and import/export your samples in a responsible manner and in compliance with local, national and international laws, noting any permits that were obtained (give the name of the issuing authority, the date of issue, and any identifying information).*
Disturbance	*Describe any disturbance caused by the study and how it was minimized.*

Reporting for specific materials, systems and methods

We require information from authors about some types of materials, experimental systems and methods used in many studies. Here, indicate whether each material, system or method listed is relevant to your study. If you are not sure if a list item applies to your research, read the appropriate section before selecting a response.

Materials & experimental systems

n/a	Involved in the study	
☐	☒	Antibodies
☒	☐	Eukaryotic cell lines
☒	☐	Palaeontology and archaeology
☒	☐	Animals and other organisms
☐	☒	Human research participants
☒	☐	Clinical data
☒	☐	Dual use research of concern

Methods

n/a	Involved in the study	
☐	☐	ChIP-seq
☐	☒	Flow cytometry
☐	☐	MRI-based neuroimaging

Antibodies

Antibodies used

Target; Fluorophore; Panel; Clone; Vendor; Cat#; Dilution (ul/100ul)
CD62L BV480 v1, v2 DREG-56 BD 566174 5 ul
CD86 PerCP-Cy5.5 v1, v2 IT2.2 Biolegend 305419 5 ul
CD27 BV750 v1, v2, ICS O323 Biolegend 302849 2.5 ul
CD19 BV570 v1, v2, ICS HIB19 Biolegend 302235 2.5 ul
CD45 Spark NIR 685 v2 2D1 Biolegend 368552 1.25 ul
CD1c BV510 v2 L161 Biolegend 331534 1.25 ul
IgM BV711 v1, v2, ICS MHM-88 Biolegend 314539 1.25 ul
CXCR3 A647 v1, v2, ICS G025H7 Biolegend 353711 1.25 ul
CXCR4 PerCP-e710 v1, v2 12G5 eBioscience 46-9999-41 1.25 ul
CCR7 A488 v1 G043H7 Biolegend 353205 1.25 ul
CD24 PerCP v1, v2, ICS ML5 Biolegend 311113 1.25 ul
CD3 BUV 805 v1, v2, ICS UCHT1 BD 612896 0.6 ul
CD11c APC-Fire750 v1, v2, ICS S-HCL-3 Biolegend 371509 0.6 ul
CD138 APC-R700 v1, v2 MI15 BD 566051 0.6 ul
HLA-DR BV650 v1, v2 L243 Biolegend 307649 0.6 ul
CD95 BV785 v1, v2 DX2 Biolegend 305645 0.6 ul
CD14 BUV805 v1, v2 M5E2 BD 612902 0.6 ul
CD23 APC v2 EBVCS-5 Biolegend 338514 0.3 ul
CD69 BUV 737 v1, v2 FN50 BD 612817 0.3 ul
IgD BV605 v1, v2, ICS IA6-2 Biolegend 348231 0.3 ul
CD21 PE-Dazzle594 v1, v2, ICS Bu32 Biolegend 354921 0.3 ul
CD38 BB515 v1, v2, ICS HIT2 BD 564499 0.3 ul
CXCR5 PE v1, v2, ICS J252D4 Biolegend 356903 0.3 ul
CD40 A532 v1, v2 5C3 Novus NBP1-43416AF523 0.3 ul
PD-1 PE-Cy7 v1, v2 EH12.2H7 Biolegend 239917 0.3 ul
IgG BV421 v1, v2 M1310G05 Biolegend 410703 0.15 ul
CD10 PE-Cy5 v1, v2 HI10a Biolegend 312205 0.15 ul
CD25 e450 v1 BC96 eBioscience 48-0259-41 5 ul
CD1d BV510 v1 51.1 Biolegend 350313 2.5 ul
ICOS-L APC v1 2D3 Biolegend 309407 5 ul
B220 Spark NIR 685 v1 RA3-6B2 Biolegend 103268 2.5 ul
T-bet APC ICS 4B10 Biolegend 644814 1.25 ul
Viability Zombie NIR v1,2 NA Biolegend 423106 0.2 ul

Validation

All antibodies have been validated by the manufacturer for use in targeting human proteins as indicated above.

Human research participants

Policy information about studies involving human research participants

Population characteristics

Population characteristics are fully described in Supplementary table 1 of the manuscript.

Recruitment

Written informed consent was obtained from all participants or, if they were unable to provide informed consent, obtained from designated healthcare surrogates. Healthy donors (n = 36) were recruited using promotional materials approved by the Emory University Institutional Review Board. Subjects with COVID-19 (n = 19) were recruited from Emory University Hospital, Emory University Hospital Midtown and Emory St. Joseph's Hospital, all in Atlanta, GA, USA. All non-healthy donor subjects were diagnosed with COVID-19 by PCR amplification of SARS-CoV-2 viral RNA obtained from nasopharyngeal or oropharyngeal swabs. Subjects with COVID-19 were included in the study if they were 18 to 80 years of age, not immunocompromised, and had not been given oral or intravenous corticosteroids within the preceding 14 days.

Ethics oversight

All research was approved by the Emory University Institutional Review Board (Emory IRB numbers IRB00058507, IRB00057983, and IRB00058271) and was performed in accordance with all relevant guidelines and regulations.

Note that full information on the approval of the study protocol must also be provided in the manuscript.

ChIP-seq

Data deposition

☐ Confirm that both raw and final processed data have been deposited in a public database such as GEO.

☐ Confirm that you have deposited or provided access to graph files (e.g. BED files) for the called peaks.

Data access links
May remain private before publication.

For "Initial submission" or "Revised version" documents, provide reviewer access links. For your "Final submission" document, provide a link to the deposited data.

Files in database submission

Provide a list of all files available in the database submission.

Genome browser session
(e.g. UCSC)

Provide a link to an anonymized genome browser session for "Initial submission" and "Revised version" documents only, to enable peer review. Write "no longer applicable" for "Final submission" documents.

Methodology

Replicates

Describe the experimental replicates, specifying number, type and replicate agreement.

Sequencing depth

Describe the sequencing depth for each experiment, providing the total number of reads, uniquely mapped reads, length of reads and whether they were paired- or single-end.

Antibodies

Describe the antibodies used for the ChIP-seq experiments; as applicable, provide supplier name, catalog number, clone name, and lot number.

Peak calling parameters

Specify the command line program and parameters used for read mapping and peak calling, including the ChIP, control and index files used.

Data quality

Describe the methods used to ensure data quality in full detail, including how many peaks are at FDR 5% and above 5-fold enrichment.

Software

Describe the software used to collect and analyze the ChIP-seq data. For custom code that has been deposited into a community repository, provide accession details.

Flow Cytometry

Plots

Confirm that:

☒ The axis labels state the marker and fluorochrome used (e.g. CD4-FITC).

☒ The axis scales are clearly visible. Include numbers along axes only for bottom left plot of group (a 'group' is an analysis of identical markers).

☒ All plots are contour plots with outliers or pseudocolor plots.

☒ A numerical value for number of cells or percentage (with statistics) is provided.

Methodology

Sample preparation

Peripheral blood samples were collected in heparin sodium tubes and processed within 6 hours of collection. PBMCs were isolated by density gradient centrifugation at 1000 x g for 10 minutes. Aliquots from the plasma layer were collected and stored at -80C until use. PBMCs were washed 2 times with RPMI at 500 x g for 5 minutes.

Instrument

Cells were analyzed on a Cytek Aurora flow cytometer (V3; 16V-14B-10YG-8R)

Software

Cells were analyzed on a Cytek Aurora flow cytometer using Cytek SpectroFlo software. Up to 3 x 106 cells were analyzed using FlowJo v10 (Treestar) software.

Cell population abundance

NA

Gating strategy

Gating strategy is provided in supplementary figure 1.

☒ Tick this box to confirm that a figure exemplifying the gating strategy is provided in the Supplementary Information.

Magnetic resonance imaging

Experimental design

Design type

Indicate task or resting state; event-related or block design.

Design specifications

Specify the number of blocks, trials or experimental units per session and/or subject, and specify the length of each trial or block (if trials are blocked) and interval between trials.

Behavioral performance measures

State number and/or type of variables recorded (e.g. correct button press, response time) and what statistics were used to establish that the subjects were performing the task as expected (e.g. mean, range, and/or standard deviation across subjects).

Acquisition

Imaging type(s) | *Specify: functional, structural, diffusion, perfusion.*

Field strength | *Specify in Tesla*

Sequence & imaging parameters | *Specify the pulse sequence type (gradient echo, spin echo, etc.), imaging type (EPI, spiral, etc.), field of view, matrix size, slice thickness, orientation and TE/TR/flip angle.*

Area of acquisition | *State whether a whole brain scan was used OR define the area of acquisition, describing how the region was determined.*

Diffusion MRI ☐ Used ☐ Not used

Preprocessing

Preprocessing software | *Provide detail on software version and revision number and on specific parameters (model/functions, brain extraction, segmentation, smoothing kernel size, etc.).*

Normalization | *If data were normalized/standardized, describe the approach(es): specify linear or non-linear and define image types used for transformation OR indicate that data were not normalized and explain rationale for lack of normalization.*

Normalization template | *Describe the template used for normalization/transformation, specifying subject space or group standardized space (e.g. original Talairach, MNI305, ICBM152) OR indicate that the data were not normalized.*

Noise and artifact removal | *Describe your procedure(s) for artifact and structured noise removal, specifying motion parameters, tissue signals and physiological signals (heart rate, respiration).*

Volume censoring | *Define your software and/or method and criteria for volume censoring, and state the extent of such censoring.*

Statistical modeling & inference

Model type and settings | *Specify type (mass univariate, multivariate, RSA, predictive, etc.) and describe essential details of the model at the first and second levels (e.g. fixed, random or mixed effects; drift or auto-correlation).*

Effect(s) tested | *Define precise effect in terms of the task or stimulus conditions instead of psychological concepts and indicate whether ANOVA or factorial designs were used.*

Specify type of analysis: ☐ Whole brain ☐ ROI-based ☐ Both

Statistic type for inference (See Eklund et al. 2016) | *Specify voxel-wise or cluster-wise and report all relevant parameters for cluster-wise methods.*

Correction | *Describe the type of correction and how it is obtained for multiple comparisons (e.g. FWE, FDR, permutation or Monte Carlo).*

Models & analysis

n/a	Involved in the study
☐	☐ Functional and/or effective connectivity
☐	☐ Graph analysis
☐	☐ Multivariate modeling or predictive analysis

Functional and/or effective connectivity | *Report the measures of dependence used and the model details (e.g. Pearson correlation, partial correlation, mutual information).*

Graph analysis | *Report the dependent variable and connectivity measure, specifying weighted graph or binarized graph, subject- or group-level, and the global and/or node summaries used (e.g. clustering coefficient, efficiency, etc.).*

Multivariate modeling and predictive analysis | *Specify independent variables, features extraction and dimension reduction, model, training and evaluation metrics.*
